# Gamifying model-based engineering: the PapyGame experience

**DOI:** 10.1007/s10270-023-01091-8

**Published:** 2023-03-04

**Authors:** Antonio Bucchiarone, Maxime Savary-Leblanc, Xavier Le Pallec, Antonio Cicchetti, Sébastien Gérard, Simone Bassanelli, Federica Gini, Annapaola Marconi

**Affiliations:** 1 Fondazione Bruno Kessler, Trento, Italy; 2 University of Lille, Lille, France; 3 Mälardalen University, Västerås, Sweden; 4 Université Paris-Saclay, CEA, List, 91120 Palaiseau, France; 5 University of Trento, Trento, Italy

**Keywords:** Model-based engineering, Education, Gamification, Papyrus

## Abstract

Modeling is an essential and challenging activity in any engineering environment. It implies some hard-to-train skills such as abstraction and communication. Teachers, project leaders, and tool vendors have a hard time teaching or training their students, co-workers, or users. Gamification refers to the exploitation of gaming mechanisms for serious purposes, like promoting behavioral changes, soliciting participation and engagement in activities, etc. We investigate the introduction of gaming mechanisms in modeling tasks with the primary goal of supporting learning/training. The result has been the realization of a gamified modeling environment named PapyGame. In this article, we present the approach adopted for PapyGame implementation, the details on the gamification elements involved, and the derived conceptual architecture required for applying gamification in any modeling environment. Moreover, to demonstrate the benefits of using PapyGame for learning/training modeling, a set of user experience evaluations have been conducted. Correspondingly, we report the obtained results together with a set of future challenges we consider as critical to make gamified modeling a more effective education/training approach.

## Introduction

The digital transition holds the promise of solutions to meet our societal, economic, and environmental challenges. But it comes with its own challenges, in particular the mastery of the complexity of systems, resulting from numerous interactions and requiring multidisciplinary approaches. In that context, there is no doubt that collective intelligence (CI) is an essential key, if not the key, to our society. Under the effect of collective intelligence, groups can master the complexity of the systems they have to invent to face tomorrow’s societal challenges. As featured in [1] CI requires facilities for knowledge sharing, problem-solving, and decision-making among individual users and groups, through tools fostering indeed interactions and collaborations. Model-driven engineering offers technologies that provide such facilities and should be considered a real asset to fully and efficiently exploit the high potentialities of CI. An important requirement at the heart of CI is the ability to communicate and share knowledge. Models and their various representations—which are the cornerstone of MDE—are powerful means of addressing this. Therefore, encouraging CI to solve complex problems raises challenges, and difficulties, and needs to develop modeling skills in future engineering professionals. But the widespread adoption of MDE technologies (e.g., modeling tools, code generators, or even formal analyzers) remains limited due to their use complexity [2, 3]. Gamification [4] is an exciting and promising way to remove these barriers and encourage all stakeholders’ commitment to a project team.

Within this paper, we present our initiative that started two years ago and is known as PapyGame.^1^ We have experimented with the gamification of software modeling tools in a real and robust modeling environment (Papyrus^2^) to reduce its use complexity. Our contribution is triple. First, we propose the complete and usable implementation of a gamified modeling environment. Second, we have asked students to use it and get their feedback to have the first evaluation of the user, learner, and gamer experiences of such an approach. Third, to design our solution, we have defined a conceptual architecture that can be used to gamify other modeling environments.

The paper presents our approach and these three contributions as follows. Section 2 presents a state of art on gamification in software engineering and modeling. In Sect. 3, we explain the dimensions of gamification we cover and the related mechanisms we have chosen. Section 4 describes the conceptual architecture we have defined and details one per one each of its parts. Section 5 reports on how each technical component of PapyGame has been implemented with regard to the previously defined conceptual architecture. Section 6 explains the evaluation of PapyGame with students from a user experience point of view. Section 7 discusses the lessons learned from our PapyGame experience. Section 8 concludes the paper and draws some perspectives.

## Related works

In general, a clear distinction exists between gamification and serious games: The former refers to the use of gaming mechanisms in non-gaming contexts (e.g., challenges, points, etc.) [4]; the latter instead regards the use of games and/or animations tailored to specific goals [5]. In this respect, the efforts described in this article are devoted to the *gamification* of *software modeling*, a specific activity of software engineering.

### Gamification in software engineering

Education and training suffer from a quick loss of students’ engagement, and this problem is even exacerbated in distance education. Several countermeasures have been tried, like mixing learning styles, partitioning courses in micro-modules, etc. Their common goal is to implicitly keep students motivated through small amounts of effort and rapid/visible progression in their study. On the same line, gamification uses gaming mechanisms for enhancing students’ engagement [6–8]. Since software engineering education is no exception, it is not surprising that gamified approaches exist for learning software engineering [9] and modeling [10].

There is a wide interest in adopting gamification solutions for supporting engagement in software modeling and production with learning purposes, e.g., students to actively participate in courses [11, 12], employees to deal with tedious tasks [7, 13–16], or programmers to learn specific languages [17–20]. This interest is also testified by the availability of hundreds of gamification development platforms that offer prepackaged templates to build-up gameful applications [21].

As a result, several gamification approaches have been proposed. Examples are the works of Cosentino et al. in [8], Calderón et al. in [22], and also [23–25]. Their common goal is raising the level of abstraction of gamification mechanisms and proposing a well-defined set of languages for designing a game, its main components, and the behavioral details.

### Gamification in software modeling

In this paper, we are especially interested in the works at the intersection of gamification and software modeling. In [9], the authors have analyzed *what particular software engineering processes have been the object of gamification*. They found out that while part of software requirements, development, and testing attracted most of the interest in the field of gamification, some crucial areas such as *Software Modeling and Verification* have been left out. A more recent survey [26] discusses several game-based and game development-based learning approaches devoted to software design. However, it confirms the scarce experimentation of gamification techniques for software design. In [27], the authors have explored the potential of gamification in the context of software engineering education. Through a questionnaire-based approach, they report the adoption of some game elements (i.e., badges and leaderboards) in an introductory software engineering course to evaluate the students’ perception of the impact of these elements on their motivation toward the course. Recently, virtual reality (VR) approaches have also been proposed as more immersive environments to edit UML models [28, 29]. Since these environment propose a radically new way of tackling modeling, they are typically able to capture the attention of students, making them suitable to implement gamified modeling frameworks for education [30].

All the research introduced until now reinforces the idea that there is a strong interest in the use of gamification in modeling and some first promising results, but they also show that previous attempts are mainly the work of modelers trying to manually create some ad hoc gamification environment for their specific experimental scenarios. A real attempt to integrate full gamification in modeling tools still needs to be included. A few exceptions are [8, 17–20], which can be seen as some first attempts at tackling the problem of gamifying modeling and in particular, addressing modeling education/learning. These authors propose partial technical solutions addressing specific learning objectives (data/process modeling, etc.).

In this respect, our vision abstracts those attempts by proposing a general approach for integrating modeling tools with a fully fledged gamification environment to facilitate the specification and deployment of all modeling games. Moreover, the game definition mechanisms distinguish various learning dimensions, notably learning abstraction, a modeling language, or a modeling tool. In turn, these target gamification scenarios raise relevant challenges pertaining to research and technical aspects of modeling.

## Our gamification approach for PapyGame

We have implemented a gamified version of a software modeling environment in order to experience and experiment such a gamification. In this section, we present the approach we have adopted for this development. The first subsection discusses the targeted population and learning objectives. Then, we present the interaction scheme we have defined between the gamified software and the learner. Finally, the last subsection provides details on the gamification elements involved in this scheme.

### Gamification dimensions in modeling

We investigate the introduction of gaming mechanisms in modeling tasks with the primary goal of supporting learning/training.

We distinguish three main learning objectives related to modeling: (1) *learning of modeling principles*, (2) *learning of a modeling language*, and (3) *learning of a modeling tool*. Despite being tightly interconnected, the mentioned learning goals are different as they entail different gaming mechanisms, and hence a different kind of support. In fact, in the first dimension the main intent of gaming mechanisms is to stimulate engagement and interest in learning, conveying both fundamental theory and best practices, e.g., on how to approach abstraction. In the second kind of learning objective, the typical learner can be considered a domain expert that needs to understand how domain concepts should be appropriately represented in the language under learning. The third dimension targets tool users: Gamification in this case could be used to gradually disclose features to a new user, to show the way of working for an experienced user of another tool, to demonstrate the meaning of new features, and so forth.

Orthogonal to learning objectives, we also consider the required/target level of proficiency, and we distinguish basic, intermediate, and advanced level of proficiency. In particular, a basic level entails the ability to complete tasks by matching the required representation needs, an intermediate level would also require the effective use of appropriate language/tool features, and an advanced level would demand the satisfaction of specific quality attributes for the produced models. It is worth noting that this distinction is critical in designing useful gamified applications: Proposing a basic-level task to advanced users could make them lose the interest in learning; and in the same way that giving too advanced modeling exercises to basic users could. Moreover, it is important to notice that the levels of proficiency require different gamification mechanisms, which in turn are based on different kinds of checks over the ways tasks are completed and the models produced. In this respect, especially when dealing with quality-related checks, designing adequate gamification mechanisms entails the development of ad hoc components for the evaluation.

We aim at proposing a solution that is expressive enough to support all the potential dimensions involved in the learning and their corresponding gamification. One way to support this is the use of the gamification design framework (GDF) [31], a set of DSLs devoted to the specification of gamified applications. More details about this framework are provided in Sect. 4.3.3.

### Learner–modeling environment interaction scheme

The value of gamification elements depends—partially—on when they are involved in the gamified activity [6, 32, 33]. Before explaining the choice of gamification elements and describing their role in the next subsection, we present the general interaction scheme between PapyGame and a learner and highlight when each element is used.

**Fig. 1:**
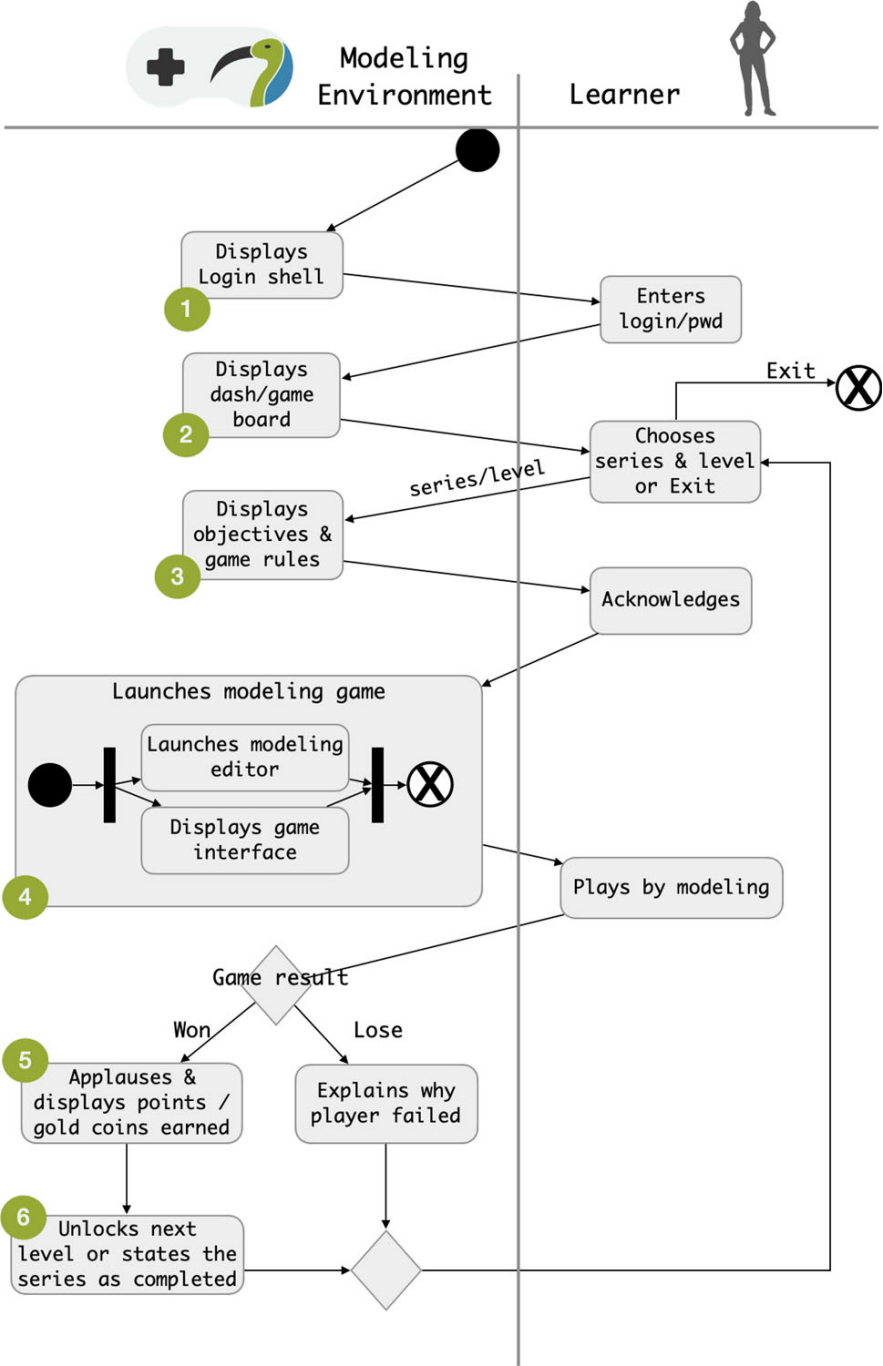
Interactions between a learner and a possible gamified software modeling environment

Figure 1 shows the interactions between a learner and the modeling environment. Figure 1 depicts an activity diagram including the actions of the learner and those of the environment.

A learner starts with a login page showed by PapyGame (step 1 in Fig. 1); after entering their credentials, they access a game board with the current status in the game and the available tasks/challenges (step 2). After selecting an available item, the learner is brought to a page illustrating the corresponding rules and objectives for the chosen game (step 3), and from there they can launch the game. Here, depending on the kind of game, the modeling environment could visualize specific interfaces together with the usual modeling editor. While playing, the learner performs modeling tasks and receives feedback until the game is over either with a win or with a loss. A win generally includes gaining points and progressing through the game stages (steps 5 and 6), while a loss is accompanied with a screen explaining the errors done during the game, and as expected the learner does not advance their game status. In both the cases, the learner is eventually brought back to the dashboard where they can decide about the new gaming step.

### Selected gamification elements

Even though, as mentioned before, there is no unique solution for gamifying modeling, we defined our approach and designed PapyGame by following specific patterns of gamification, including game elements and mechanisms, as summarized in Table 1. According to the formal framework by Deterding et al. [4] and the taxonomy proposed by Toda et al. [34], we integrated the following elements concerning the game interface design: *Acknowledgement, Level, Point, Progression* for the performance category; these elements have been chosen because they function as feedback in the learning domain, providing online information on the outcome of tasks and giving an overview of the users’ journey within the tool [35]. Overall, these elements provide important feedback that signals a player’s success [36]. Starting from points, users can level up, getting feedback on progression and getting acknowledgments. *Narrative* for Fictional; it can have different interpretations [37], is mainly described as the order of events as they happen in the game, through the user experience [34]. The main efficacy of narrative mechanics is to enhance users’ immersion and enlighten them to steadily move toward their final goals [38, 39]. *Objective* for Personal; this element is crucial in the design of gameful systems in the educational domain; in fact, there are many reasons that can drive the implementation of gamified elements. For instance, Tondello and colleagues [40] identify three main goals in gameful systems: to accomplish a specific result (outcome goals), do well by one’s own performance standards (performance goals), and learning new skills (process goals). Hence, the objective element provides the player with an end, or a purpose to perform the required tasks [34], providing also control in the design phase of the steps to arrive at the final goal, thus allowing for customization of the route according to the final goal. *Competition, Reputation* for Social; these elements reflect the social component of the gameful systems. The competition element is often linked with increased performances [41, 42]. However, the use of the competitive component should be applied carefully because its misapplication, especially when paired with the use of leaderboards, can have a detrimental effect on users’ motivation [43], while when applied in a moderate way, competition stimulates learning and provides a playful approach [44]. In our case, we therefore decided not to use a leaderboard to provide progression feedback to users, as its combination with competitive, and even reputation, elements could have negative effects on motivation. The reputation element is often related to the competitive environment, and it is composed of titles that the learner may gain and accumulate within the environment [34]. In PapyGame, this element creates a hierarchy in the environment and, at the same time, provides additional feedback to the users about their performance, since it is related to their in-game results. Overall, reputation element is working as an extrinsic motivator [45] and can help in enhancing users’ motivation toward the activity.

**Table 1:** Gamification mechanisms used in PapyGame

Category	Performance	Fictional	Personal	Social
	Acknowledgement	Narrative	Objective	Competition
Elements	Level			Reputation
	Point			
	Progression			

Many other elements would have been interesting, such as *Time Pressure*, *Cooperation*, and *Story Telling*. But we preferred to get a reduced set of elements in order to more easily assess—in future works—the influence of each one. Moreover, we consider the elements we have selected to be a minimal but sufficient basis and appear to be the most common elements of gamification (although we do not have any studies on this last point).

By recalling the interactions illustrated in Fig. 1, we addressed the elements related to the performance category by creating points, levels, and a concept of progression between the levels in the game. Moreover, the game includes special prizes like gold coins as a form of acknowledgement. These elements form also the base for the ones related to other categories, like the objective for the personal category or competition and reputation for social. Narrative elements are supported both by means of the progression through the levels, and also within single games. In particular, we allow multiple game styles and propose two concrete variations for PapyGame, namely Hangman and model On Your Own (OYO). For the former, a fictitious stick man gradually appears as the modeler makes mistakes during the assigned task, so that once it is complete the game is over with a loss; for the latter learners are left alone working on their solution, which they submit once they consider it as complete. In this case, depending on the quality of the submitted solution, learners gain (gold) coins and advance levels when winning.

At this point, it is worth remarking that while the “backbone” of PapyGame is fixed, e.g., game board, login, levels, etc., the gaming interfaces like Hangman or OYO are realized in an extensible way through an abstract container for games. In this way, it is possible to create and easily plug-in new games, e.g., for specific levels.

## Conceptual architecture

The main goal of our work was to study in real conditions the gamification of software modeling activity. The main concrete objective was then to have a concrete gamified modeling environment (Papyrus). At the same time, we aimed at facilitating the gamification of other modeling environments.

The greater the number of tools integrating our approach, the easier it will be to learn how to model. We designed the outlines of PapyGame through a conceptual architecture whose components support the different aspects of our gamification approach. The resulting abstract architecture can then serve as a specification for future implementations.

**Fig. 2:**
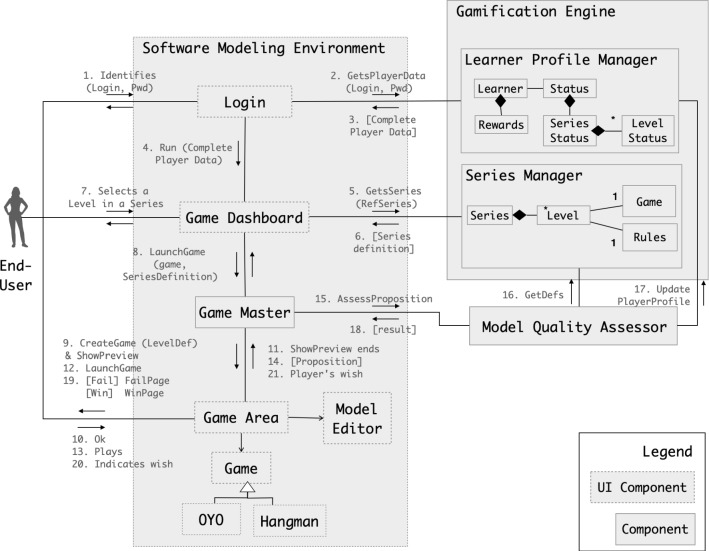
Conceptual architecture

Figure 2 illustrates all the components and their related communications through a UML collaboration diagram. The components are distributed between the Software Modeling Environment (SME) and the Gamification Engine (GE). All the components that interact with the learner are located in SME: the *Game Dashboard* and its *Login* facility, the *Game Master* and the *Game Area*. The *Game Master* orchestrates the flow of PapyGame players within the gamified modeling tool. It is never in direct contact with the players, but triggers the mechanisms of gamification from their intent to start a level the end (the success or the failure) of a level. The *Game Area* component launches games and connects them with the *Model Editor* of the SME. The *Gamification Engine* is in charge of gamification logic as described below and contains the *Learner Profiles* and *Series managers*. The *Model Quality Assessor* is the only component which is not in one of the two main parts of our architecture. The underlying objective of this position is that it can be easily outsourced.

**Fig. 3:**
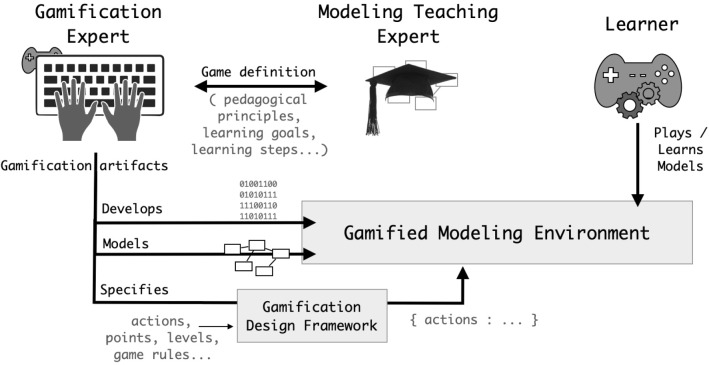
Series definitions and stakeholders involvement

From an initial definition of a series of games (see Fig. 3) and the way they interact with the profile learner, the architecture intends to allow running full-fledged gamified modeling environment executing the series.

In this section, we start by describing the different stakeholders and how they participate in gamification. We then give an overview of the data structures that are used to define the different artifacts of game, learning, progress, etc.

Section 4.3 is of particular importance, as it contains a detailed explanation of each component, as well as their involvement in performing specific activities or supporting gamification aspects. The scenario underlying in Fig. 2 is detailed at the end of this section.

### Framework stakeholders and related concerns

As illustrated in Fig. 3, our approach caters to three principal stakeholders: the (i) *Gamification Expert*, who designs and develops appropriate gamification artifacts (i.e., game elements, mechanics and dynamics) in line with generic learning goals; the (ii) *Modeling Teaching Expert* (e.g., Teacher, Methodologist, or Tool Vendor) that in collaboration with the gamification expert defines the game including the expected pedagogical principles, the global learning goals, and the suitable progression of learning steps with their intermediate learning goals; and (iii) *Learner*s (i.e., Student, Language Expert, or Software Developer) that use the gamified version of the software modeling tool to learn modeling with a given tool or even enhance their modeling expertise level while gaming.

Typically, a *Modeling Teaching Expert* specifies the modeling game defining the concrete learning objectives that shall be met at the end of a corresponding process. A *Gamification Expert*, based on the game specification, proposes a design including specific game elements to augment the target modeling tool. This phase is expected to be iterative and serves the definition of the various game levels matching the various expected progression learning steps, the specification of corresponding challenges, and the measures to concretely evaluate how users are performing with their tasks.

### Data structures

There are two important data structures in our architecture. The first one refers to the learning paths and the narrative aspect of gamification with levels, progression, points, etc. We have defined for this a system based on *Series*. A series can be seen as an adventure or a challenge made of several steps. A step is called here a *Level*. A level is defined by a statement indicating a diagram fragment to build. The level also indicates the game used for this level as well as the rules to respect (some coming directly from the game), the conditions of success, and the points accumulated (golden coins and experience points).

### Architecture components

#### Software modeling environment

In our approach, games have to be implemented within a *Software Modeling Environment* so that the learner can use its *Model Editor* to achieve the goal as prescribed by the game rules. We define a *Game Area* component where each game takes place and can interact with the learner. This area is connected to the *Model Editor* in order to provide games with access to it.

For an implementation of our approach in a given modeling tool, there will therefore be an abstract interactive component *Game* that will define four activities in an abstract way, as shown in Fig. 2. Here are the names of these activities and what they consist of:

1. Presentation: the game presents the level statement to the learner as well as the rules to be followed.
2. Game: this is the activity where the learner actually plays and so defines a particular expected model.
3. Success: the game informs the learner that they have won and the points they get back.
4. Failure: the game indicates to the learner that they lost and proposes them either to try again or to go back to the dashboard.

Even if Game is an abstract component, it can propose a template for each page so that—each level respecting a display style—the learner is always in the same graphic atmosphere.

#### Game dashboard and login

The *Game Dashboard (GD)* interactive component is the learners’ entry point of the proposed Gamified Software Modeling Environment. After the learner logs to the game framework via the *Login* interactive component, the GD allows them to access to the various available games, and to check their *games status* (i.e., history, achievements, and progresses). It also enables them to personalize their *learner profile* with new avatars, expertise levels, etc. All this information may be used by the *Game Master* component to adapt dynamically game scenarios or difficulties to end-user features and experiences.

#### Gamification design framework and gamification engine

We previously mentioned game-like elements such as awarding points or submitting challenges in order to keep users’ involvement in certain activities. We used the *gamification design framework* (GDF) to define these elements. GDF follows the principles defined by Morschheuser et al.[46] for the gamification software approaches. GDF thus proposes an approach able to raise the level of abstraction of gamification mechanisms and to provide a domain-independent solution for the design and development of gamified applications. GDF provides a modular approach that can be customized for different gameful systems and reflect a specific gamification process. It adopts reactive computing models, as advocated in [47]: The logic of a game is typically expressed as a set of rules that predicate on learners current state and are fired in response to the incoming events, i.e., gamifiable actions. Rules can modify the game state, and also fire additional events that may trigger chains of further game rules.

Thanks to GDF, we specified the main components that will be managed by the Gamification Engine (i.e., actions, points, levels, etc.) and their behavioral details (i.e., game rules). These rigorous definitions allowed us to properly implement the PapyGame components. In the GDF context, the preferred hypothesis for this implementation was to feed a gamification engine with the specifications. We used the *Gamification Engine* (GE) associated with GDF and deployed our specifications on it to *run the game*. But, any gamification engine would have been suitable. There are just some features the engine has to provide. First, it shall be in charge of launching games and executing their logic, advancing the *Game Status* for all learners in all executing games, and persisting each game state. Next, it shall store the description of series and their levels, for example in a dedicated *Series Manager* component. Finally, it shall be also in charge of providing software access to the *Learners Game Status* and, in general, to any information of the game state of all learners. This access can be used for example by the Dashboard.

#### Game master

As it is typical for role-playing games, the gamified system proposed in this vision owns a specific component dedicated to *monitor* the game execution called the *Game Master*. This latter receives notifications and/or propositions from games inside the *Software Modeling Tool* and requests the *Model Quality Assessor* component to evaluate user solutions while enabling GE to track user’s *game actions and status*. Consequently, depending on the game strategy, the learner experience, and possibly some other external factors, the Game Master can decide to adapt the game scenario (e.g., to speed-up or slow down the game dynamics) or even decide to assist the gamer in order to help her/him to achieve game goals.

#### Model quality assessor

The *Model Quality Assessor* component supplies two services. Firstly, it is used to assess if the learner has met the goal of the game. This is achieved by comparing the final model artifact with a reference result, and if they match, then the game is won; otherwise, it is lost. In some specific cases (game), the comparison might be not binary (i.e., won or lost). In fact, sometimes it is possible that one given modeling problem may be solved by different, equally good, model solutions. In other cases, some solutions are better than others. As a consequence, the *Model Quality Assessor* shall be able to measure the quality of a solution such that depending on the quality level, the learner will gain more or fewer points.

The second service provided by this component is related to game progression: The *Game Master* may need to know what is the current status of the game (in our case, the status of model artifacts) in order to monitor the learner progress and decide whether, for example, to help the learner or if to modify the game to match better a learner profile.

### Simple scenario

As mentioned above, Fig. 2 illustrates how all components collaborate in a typical running example.

Here is a simple example of the path of a player within the application:

1. The player enters their credentials via the *Login* component displayed in the modeling tool.
2. The Login component sends a request to the *Gamification Engine (GE)* for validation.
3. For this scenario, we assume that the credentials exist and are correct. The GE performs the validity check and responds to the request from the *Login component* with the player’s nickname, points and progress in the different levels.
4. The *Login* transmits this data to the *Dashboard* to be displayed with the player’s profile.
5. At startup, the *Dashboard* retrieves the available series to play from the *Series Manager*.
6. The available series are displayed in the *Dashboard*. The system is now idle until the player performs an action.
7. The player clicks on an available level of a displayed series.
8. The *Dashboard* notifies the *Game Master* that a game should be launched with parameters such as the type of the game or the level to play.
9. The *Game Master* initializes the game preview and displays it in the *Game Area*. This preview explains the rules of the game, but also specific information for the level to be played.
10. When the player is ready to start, they click on a button, which is handled by the game view in the *Game Area*.
11. The information is transmitted to the *Game Master*.
12. The *Game Master* initializes the in-game mechanisms and views and display in the *Game Area*.
13. The player now models to reach the goal of the level.
14. The proposal mechanism depends on the game. For instance, with On Your Own game, the player estimates that the model is ready to be evaluated and clicks on a button to send the model for evaluation.
15. The *Game Master* is notified. It is responsible for parsing the model to a JSON format, and to send it to the *Model Quality Assessor*. This component uses different assessment mechanisms (based on the type of the game) to estimate the validity of the proposal.
16. We assume that the player’s proposal is correct. To compute the rewards, the *Model Quality Assessor* retrieves reward rules from the *Gamification Engine* and calculates the amount of gold coins and experience to be granted to the player.
17. The profile of the player is updated with the new rewards.
18. The *Model Quality Assessor* responds to the *Game Master* with the success/failure information and the potential rewards.
19. Accordingly, the *Game Master* shows the game success or game over view in the *Game Area*.
20. The player decides to retry if they failed or decide to resume to the *Dashboard*.

## PapyGame

**Fig. 4:**
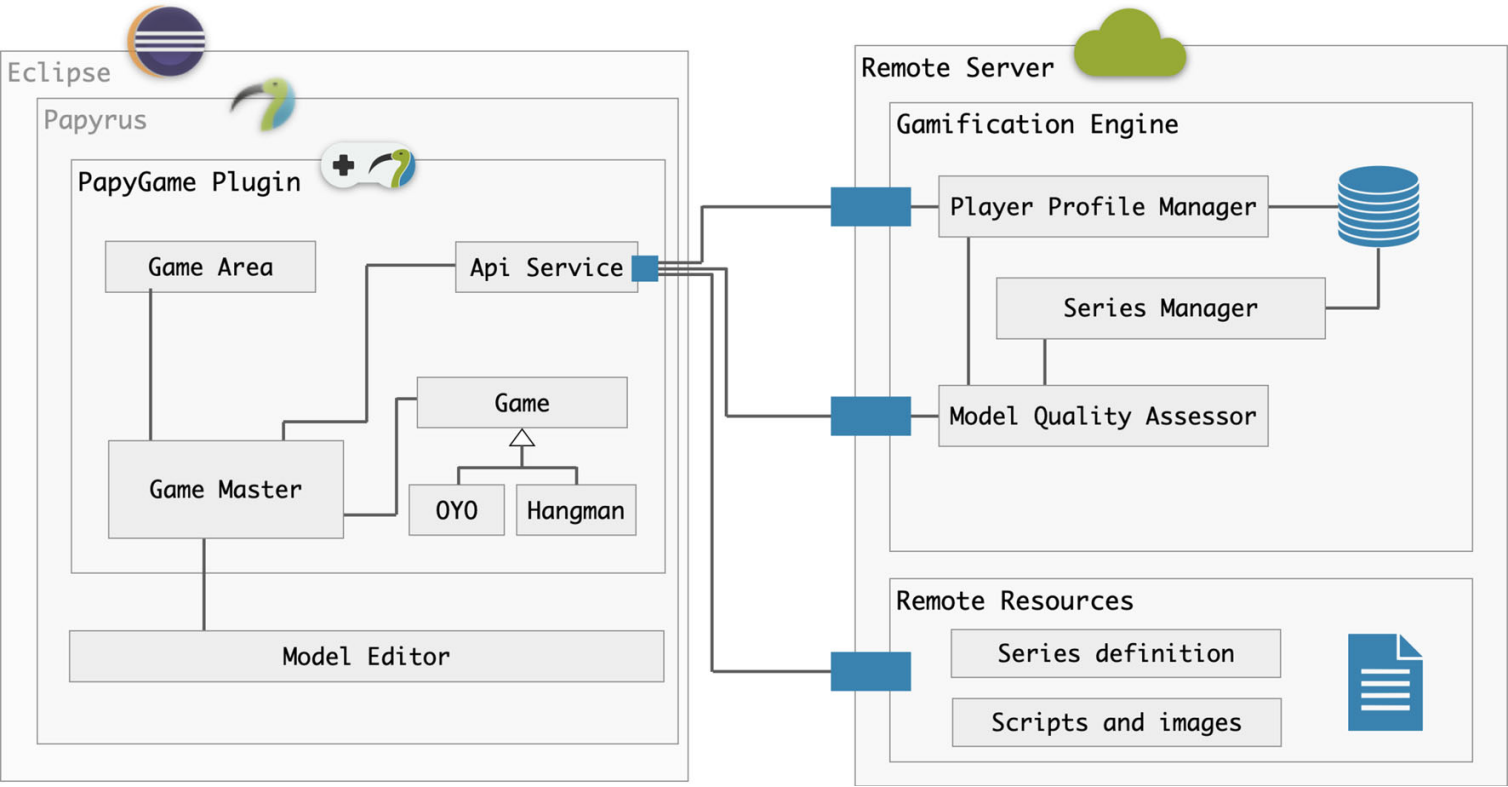
PapyGame technical architecture

PapyGame is a plugin^3^ for the modeling tool Papyrus developed by the CEA.^4^ The objective of this plugin is to gamify the learning of modeling by integrating a game view within the editor. Thus, students can learn to model by playing within a formal UML modeling tool, used by many companies. In this section, we describe the technical architecture of PapyGame which is based on the conceptual architecture presented in Sect. 4. We present how each component of the conceptual architecture has been technically implemented and provide screenshots of the different views of PapyGame. Figure 4 shows the general architecture of PapyGame.

### Server side: the gamification engine

The *Gamification Engine* (described in Sect. 4.3.3) is implemented as a standalone application deployed on a remote server. It allows to implement each key concept of the GDF [31] through user interfaces accessible from a web browser. For PapyGame, the Gamification Engine plays three main roles: *Learner Profile Manager*, *Series Manager*, and *Model Quality Assessor*. The server on which the Gamification Engine is launched also provides the client with useful remote resources (JSON files, images, JavaScript scripts).

#### Player profile manager

In order to implement gamification mechanisms related to progression, experience, rewards, and more generally to a player’s profile, the *Gamification Engine* provides a set of administration interfaces and endpoint APIs to manage the player database. The Gamification Engine natively manages the notion of player profile by integrating the nickname, the current level of the player, and the storage of the current level of the rewards obtained by the player. However, there is no system for keeping track of the player’s game history, nor the history of experience and award acquisition. To keep track of these histories, we use a *customData* field included in each player profile, in which we store the history of wins and awards in JSON format. User profiles can be created, read, updated, or deleted from REST API endpoints exposed automatically by the Gamification Engine. These endpoints are called by (i) the PapyGame client when logging in or displaying the dashboard or (ii) the *Model Quality Assessor* component of the Gamification Engine when receiving a completed game request from the client.

#### Series definition and series manager

**Fig. 5:**
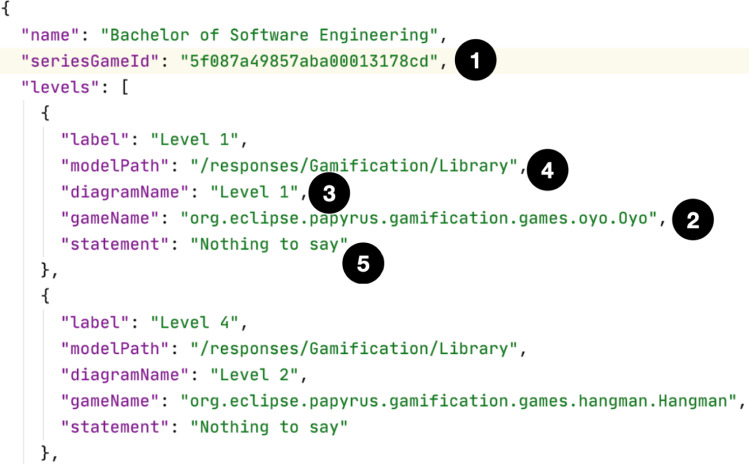
JSON description of a series

In the current implementation of PapyGame, the definition of a Series is done in two distinct steps. First, the Modeling Teaching expert has to design the different levels that will compose the game series by writing their instructions, by choosing the associated games, by defining an identifier for them, and eventually by associating a UML starting model if a comparison or an automatic generation of the exercise is planned. The series and the levels that compose it are completely defined in JSON as presented in Fig. 5. The resulting JSON file is then made available on the resource server, but is not part of the Gamification Engine. Each level connects to the Gamification Engine rules thanks to a specific id (1) held by the series, references a game (2), a source diagram (3) from a source model (4), and features textual instructions (5).

In a second step, the Gamification Expert has to define the conditions of success or failure, as well as the mechanism of reward or experience attribution. These rules are defined in the Open Source DROOLS rule engine [47] in the Gamification Engine, which provides the action mechanism. For PapyGame, we use one action per series and detail the rules for each level through conditional branches. The rules for computing experience and gold coins (the two reward concepts used in PapyGame) are functions of (i) the time taken to complete the level, (ii) the number of errors, (iii) the number of attempts if applicable. Each action created can be called through an endpoint API in GET or POST, which is called by *the Model Quality Assessor* component.

#### Model quality assessor

The *Model Quality Assessor* bridges the client requests issued by the *PapyGame* *plugin* containing the player diagram data and the rules of each level defined in the *Gamification Engine*. For PapyGame, it is the internal action mechanism of the Gamification Engine that plays the role of Model Quality Assessor by executing the rules when a request is received. This module accepts POST requests containing the player’s ID, the time taken to complete the level, the content of the diagram parsed in JSON, and possibly the number of errors or unsuccessful attempts. The number of errors can be calculated directly from the client or in the Model Quality Assessor depending on the type of game being run. When executing the rules, the Model Quality Assessor evaluates whether the attempt is a failure or a success and, if so, updates the player profile (progress and history) thanks to the end point exposed by the *Player Profile Manager*.

In our approach, we used two main methods for assessing the validity of the submission of a player. The first one is the *direct comparison* of tagged elements: the Gamification Expert first creates a diagram with elements that are expected in the submission of players. By using tags in the names of elements, the Gamification Expert is able to specify what should be checked for exact matching during the evaluation of a submission. For instance, naming a class attribute [tv] age automatically triggers the check for exact matching of the *type* and *visibility* of the attribute *age* between the source diagram from the Gamification Expert and the submission of the player. This matching is made *locally* in the embedded code of the modeling tool.

**Fig. 6:**
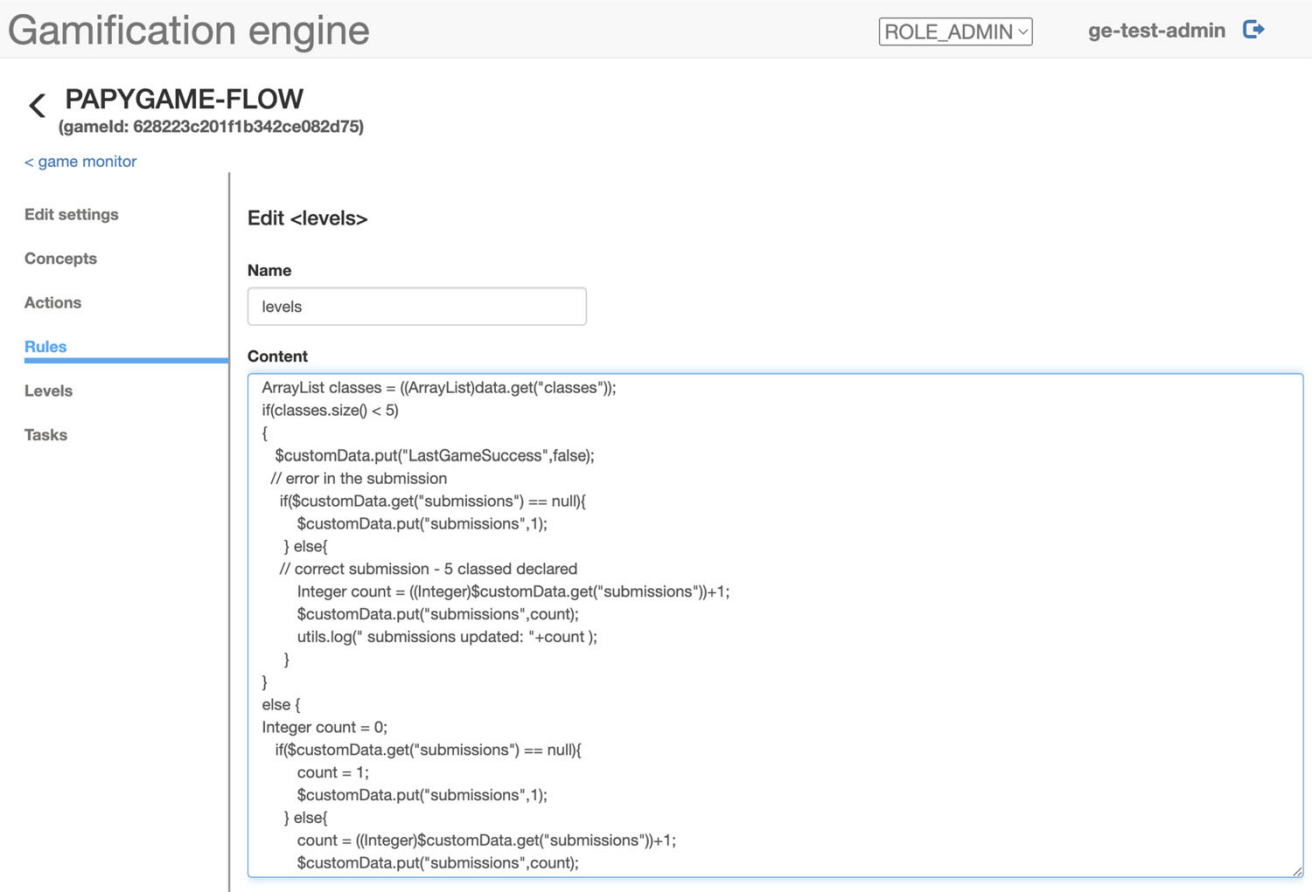
A DROOLS rule of PapyGame in the gamification engine

The second evaluation method relies on the extraction of features from the diagram. The Gamification Expert specifies, for instance, the minimum (or exact) number of attributes, or the name of minimum attributes of a submission in order to pass the level. Such numbers or lists—that we call *features*—are then checked on the *server side*. During this process, the code embedded in the modeling tool is responsible for parsing the diagram in the editor to JSON, and to compute the value of specific hard-coded features. In the current version of the gamified environment, the rules can then only use a limited set of features. Figure 6 shows an example of DROOLS rules checking the value of features about the *number of classes* in the diagram. The player’s submission is expected to have at least 5 classes declared.

### Client side: bringing games into Papyrus

In this section, we describe the architecture we designed in Papyrus to create a modular game execution environment. We present the role of each component of the client, including the *Plugin*, the *Game Master*, the *Game Object*, and how they interact with each other.

#### The PapyGame plugin

Papyrus relies on the Eclipse environment due to its distribution as an Eclipse plugin. Thus, it enables developers to augment it with new modules, by retrieving the proper services and listening to dispatched callbacks. The PapyGame client follows this logic and is distributed as an Eclipse plugin written in Java, that is available for download and install in its own update site.^5^ From a client architecture perspective, the plugin is responsible for providing the technical bricks which tie logical components together. This includes creating the *Game Area* inside Papyrus, and initializing the *Api Service*. The *Game Area* is a view container which allows other components to display their own views. In Eclipse, we chose to use a Browser component for the Game Area, so we could use web technologies such as HTML, CSS, and JavaScript to create responsive and user-friendly UI. The *Api Service* is in charge of handling network requests to the server. It relies on the use of Retrofit 2, which remove the writing of boilerplate networking code, and automatically parses the requests in JSON with the use of Gson.^6^ Asynchronous communication with the server is handled with RxJava 2.^7^

#### The game object

In PapyGame, a *Game* is a set of Java classes and HTML/CSS/JavaScript files which describe the mechanics of the behavior of a game. Each Game contains four views: the Preview View, the InGame view, the GameSuccess view, and the GameOver view. Technically, each view is associated with one Java class and one HTML file. (Styles and scripts can be added to the HTML file.) The Java classes of the views contain the internal behavior of the Game, e.g., how to react to a input user event, and define an entry point to be executed by the *Game Master* component. While the Preview, GameSuccess, and GameOver views are mainly static screens to display information, the InGame view is often dynamic, reacting to the modeling progress. This InGame view is also in charge to inform the Game Master when the game should stop, and the remote server should be queried.

#### The game master

The *Game Master* has the role of coordinating the different components. It receives the updates from the events in the *Model Editor*, the click interactions from the *Game Area*, and is connected to the *Api Service* to query the server when required. The Game Master has handled three main interaction sequences: (i) the login phase, (ii) the dashboard phase, and (iii) the game phase.

**Fig. 7:**
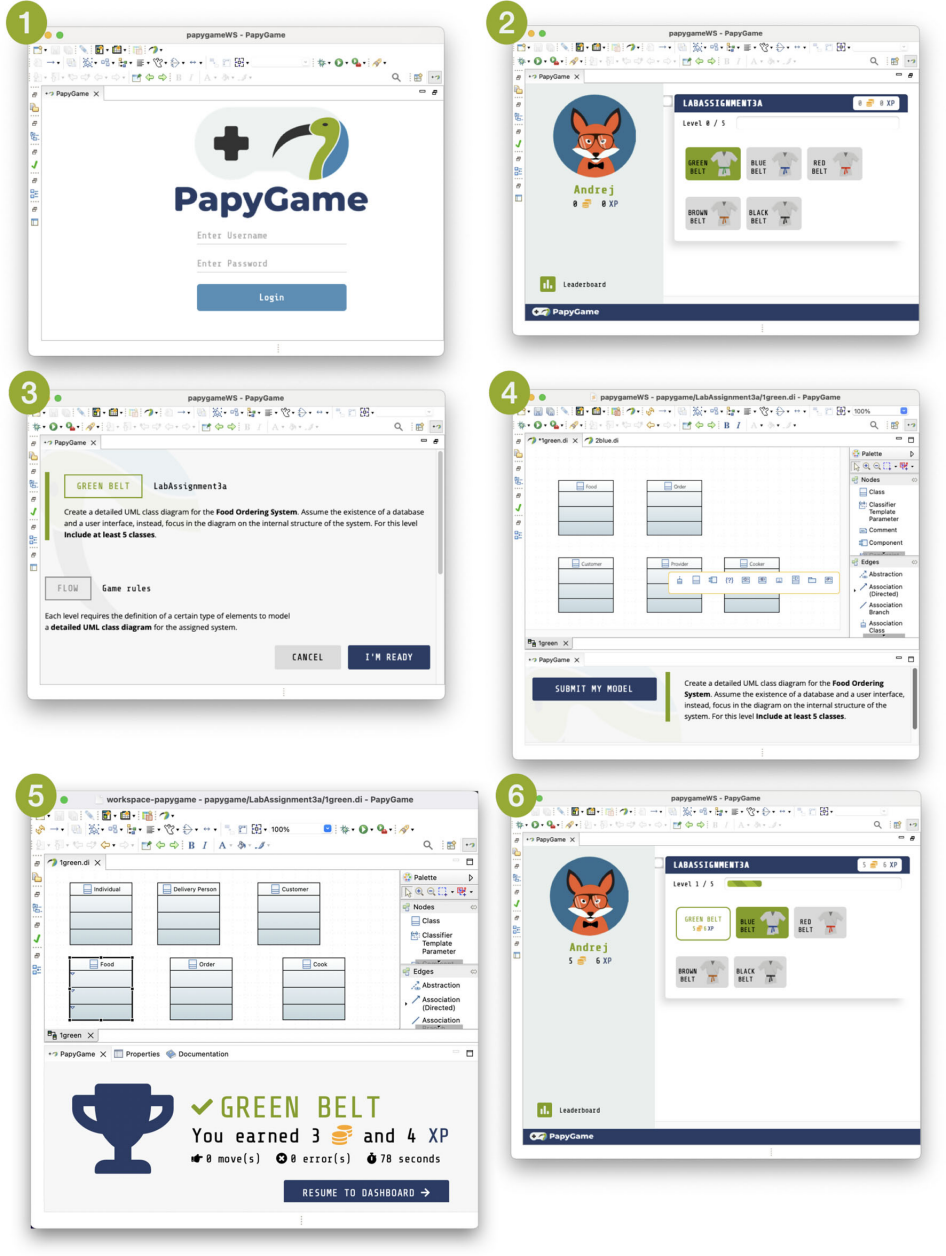
User interfaces of PapyGame

*Login phase* In order to associate players to their stored profile, PapyGame asks them to create an account. This is achieved directly from the login screen (Fig. 7  $\textcircled {{\textbf {1}}}$) by choosing a username that is available, and to type a password. If the username is available and no account exists with this username, then PapyGame shows a *confirm password* field. After typing the password confirmation, the account is created and the player is automatically logged in. During this sequence, the Game Master listens to the button clicks, asks the Api Service to query the Player Profile Manager, controls the behavior of the Login View in the Game Area including displaying fields and progress bars, and eventually switches phase to enter the Dashboard phase. When the account already exists, players just have to enter their credentials to access the dashboard.

*Dashboard phase* When the Game Master initiates the Dashboard phase, it first asks the Api Service to query the server to retrieve (i) all series available and (ii) the fully loaded player profile. Once received, it displays the series, the progress history, and the player profile information in the Dashboard View that is loaded in the Game Area, as presented in Fig. 7  $\textcircled {{\textbf {2}}}$. The Game Master stays idle until the player clicks on one available level. This triggers the launch of the Game phase, which starts with the Game Preview View.

*Game phase* The Game phase starts when the player clicks on an available level from the Dashboard View. The Game Master is then responsible for switching the view in the Game Area to the Game Preview View of the game associated with the selected level (Fig. 7  $\textcircled {{\textbf {3}}}$). Then, the Game autonomously listens to all user input events on its views. When players are ready to start, they click the button on the Preview view, and the Game asks the Game Master to switch view to the InGame view (Fig. 7  $\textcircled {{\textbf {4}}}$), which, in turn, behaves autonomously. The Game Master is idle until the Game requests the end of the in-game phase. Then Game Master then collects information provided by the Game to query the remote Model Quality Assessor to update the player profile and to know whether it should display the GameSuccess or GameOver view (Fig. 7  $\textcircled {{\textbf {5}}}$). From these two views, the player can ask to resume to the Dashboard (Fig. 7  $\textcircled {{\textbf {6}}}$). When this event is triggered, the Game asks the Game Master to switch view to the Dashboard, to initiate a new Dashboard phase.

### Available games

Thanks to its modular architecture, it is easy for gamification experts to create new games to add to PapyGame. Yet, for the purpose of this implementation and the experiment we conducted, we implemented two games which can be used for any level: the *Hangman*, when a new part of the man drawing is added with every wrong answer, and the *On Your Own* (OYO), when the student executes the exercise with no help.

**Fig. 8:**
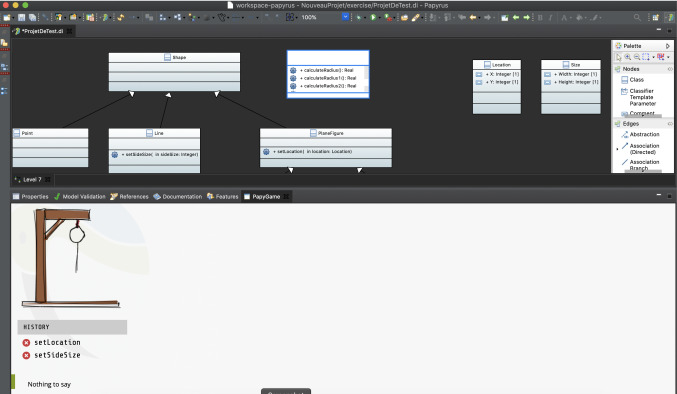
PapyGame Hangman game example

Figure 8 shows an example of the Hangman game. It presents the associated UML diagram containing a set of classes connected with the generalization relationship. The goal of this level is to help players/students to associate the right attributes and operations to the right class in the hierarchy. This is done using a drag-n-drop facility. An incorrect user selection (moving an operation into a class that is not the one that should contain it) adds a part of the hangman’s body (lower part of Fig. 8). If the players manage to place all operations correctly without the body of the hanged person being completely displayed, they win. If the hangman’s body is completely displayed, players lose, and the next level stays locked. As a consequence, they will have to play the level again.

## Evaluation

In order to accurately evaluate PapyGame, we designed two different evaluation procedures. In the first one, we developed an ad hoc user experience questionnaire, which allowed us to gather information about the user experience, the application procedure, comments about PapyGame strengths and weaknesses, and suggestions regarding the software improvement. In the second one, we used a modified version of a preexisting questionnaire in order to consolidate first results and to get an overall evaluation of the software quality according to the user/learner/gamer experiences.

### PapyGame preliminary evaluation

#### Rationale

In developing PapyGame, we made rational design choices that come from serious and validated works. Thus, to evaluate our approach exhaustively, we preferred to firstly evaluate our work with a user experience questionnaire. An ad hoc created questionnaire (Table 2) was presented to users with an initial part aimed at the collection of generic data (age, gender and country), a part to differentiate between the two groups (*“What is the software you used / are using?”*) and a final part to give opinion about what is working well and what needs to be changed in the software. Then, the questionnaire evaluates several aspects related to the user experience (i.e., motivation, engagement, attractiveness, learning perception) and to software usability and perceived efficacy.

**Table 2:** User experience questionnaire

Dimension	Item
Attractiveness	I find this software enjoyable
	I find this software annoying
Efficacy	I think this software has slowed down my learning
	I find this software efficient
Usability	I find this software complicated
	I think this software is easy to learn
Motivation	I enjoy learning modeling
	I find this program stimulating
Engagement	I put enough effort into learning modeling
	I didn’t try very hard to do well in the tasks
Learning perception	I am able to use the skills learned in this course outside of class
	I feel more self-reliant as the result of the content learned in this course

#### Participants and procedure

A total of 14 students from the university of Lille (France) participated to the study (mean age = 21.14 ± 1.87 years old; 4 users were females and 10 were males).

The responses were on a five-item Likert-type scale (disagree = 1; agree = 5)[48]. After two weeks, users had to fill-in the user experience questionnaire attached to the software.

#### Questionnaire results

Results were calculated using the statistical software RStudio^8^ (v. 2021.09.0). Table 3 presents an overview of the results. They suggest medium/high values to positive items, and low values for those items related to negative components (“*I find this software annoying*,” “*I think this software has slowed down my learning*,” “*I didn’t try very hard to do well in the tasks*”).

**Table 3:** Group descriptives

Item	Mean	SD
I find this software enjoyable	3.429	1.284
I am able to use the skills learned in this course outside of class	4.143	0.663
I enjoy learning modeling	3.143	1.292
I find this software stimulating	3.357	1.008
I think this software is easy to learn	3.071	1.072
I feel more self-reliant as the result of the content learned in this course	3.429	0.646
I find this software efficient	3.929	0.997
I put enough effort into learning modeling	3.571	1.158
I find this software complicated	3.214	0.975
I find this software annoying	2.643	1.336
I didn’t try very hard to do well in the tasks	2.929	1.072
I think this software has slowed down my learning	2.071	0.997

We analyzed also the responses to the open questions “*The aspect I appreciated the most*” and “*The aspect that disturbed me the most*.” The most appreciated aspects of the software were the animation of the software, the game aspects, the usability, the gamification elements used (money and levels), the colored belts, the graduation of the software, the efficacy. The most disturbing elements were the lack of information, the design components (dimension of buttons, localization, typing, scrolling), the complexity and problems related to the Eclipse environment.

To summarize, this first use of PapyGame suggests an overall positive result in each domain of the user experience questionnaire, especially in the efficacy perception and learning perception domains.

### PapyGame overall evaluation

#### Rationale

The first evaluation provided encouraging results regarding the user experience of PapyGame. In order to strengthen the demonstration of these benefits, we performed a second evaluation, still focusing on the user experience, but taking into account the aspects related to the game principle. First, obtaining the same results in terms of user experience by using two different tests has a positive impact on the validity of the feature under study, as mentioned in the *convergent validity* theory [49]. Regarding also the “methodological triangulation” [50, 51], it is important to increase confidence in having captured the concept [52]. Second, there is evidence of differences in the evaluation of gamified software depending on learners’ gaming habits [53]. So, through this second evaluation we aimed at increasing the validity of the scale we adopted in the first evaluation, and investigating any existing difference between gamers and non-gamers.

For this, we used the MEEGA360^9^ scale [54] which is an evolution of MEEGA+ scale[55]. Based on a rigorous systematic literature review [56], MEEGA+ is a model for evaluating the quality of educational games. It aims at assessing fun, perceived learning, usability and user experience of a software. It uses a full but limited number of items (35). Moreover, the goodness of the software can be assessed by calculating the total score of the scale. The MEEGA360 [54] brings more reliability in the calculation of the overall score, if compared to the MEEGA+, due to the introduction of reverse items (excluded from the calculation of the overall score), which allows checking for biases and patterns in users’ answers. Also, the introduction of items assessing users’ motivation, and the elimination of confusion or unnecessary items, makes the MEEGA360 more suitable for our context.

#### Participants and procedure

A sample of 16 students from the University of Trento in Italy participated in the experiment (age between 18 and 26 years old, 2 females, 14 males). In addition, participants were divided into two groups based on their responses about their gaming habits in order to analyze the influence of gaming habits on the perceived quality of PapyGame. The first group (gamers, n = 8) consisted of students who played video games regularly (daily or weekly), while the second group (non-gamers, n = 8) consisted of participants who played video games occasionally (monthly, rarely, never).

Students first used PapyGame and then completed the MEEGA360 scale to assess the overall quality of the gamified software. Responses were given on a five-point Likert-type scale (from 1 = disagree to 5 = agree).

To evaluate the overall goodness of the gamified software PapyGame, we presented students with the MEEGA360 scale.

#### MEEGA360 overall quality score

The analysis was performed in Jamovi^10^ (v. 1.6.23.0). We calculated the mean of the MEEGA360 total score of the 16 participants and compared the results with the three ranges related to the goodness of the software (low quality $\theta$ < 42.5; good quality 42.5 $\le$ $\theta$ < 65; and excellent quality $\theta$ $\ge$ 65). The result (mean = 58.49 ± 4.46) shows that PapyGame lies in the middle range, indicating that the software achieves an overall good quality.

In addition, we compared the total MEEGA360 scale scores between the gamer and non-gamer groups. We decided to perform a t test for independent samples. The results (t test, t = 1.51, p >.05, d =.76) show that there is no significant difference in the perceived quality of the gamified software and the user experience between the two groups.

Since the goal of gamified solutions is to satisfy experts and novices users alike while addressing deeply held pedagogical assumptions [57], addressing the existing differences in the user experience according to the video game expertise [53], the data suggest that the software succeeds in the difficult goal of being perceived in a similar way according to the users’ video game experience.

Overall, the results indicate that PapyGame achieves a satisfactory level of quality and that the gaming habits of the participants do not influence the perceived quality of the software.

### Analyses discussion

In the first applications, PapyGame showed an overall positive evaluation from the users, which evaluation was consistent for both questionnaires used, showing convergent validity. While the tool showed adequate levels during user experience analysis, it also presented some disturbing elements, including elements of UI, the information provided, and the complexity and problems related to the Eclipse environment. We expect to fix those elements soon. Moreover, users who participated in the evaluation, all belonged to category *learning of a modeling language*, presented in Sect. 3.1, thus it is not possible to provide generalization on the utility of PapyGame on the other categories of modeling students (i.e., *learning of modeling principles* and *learning of a modeling tool*).

In order to present a more detailed evaluation, it is necessary for the future to evaluate some elements specifically, including a detailed analysis of usability, and the level of fun, and comprehensively analyze the effectiveness of the tool by correlating user results with external outcomes (i.e., results to exams). Moreover, in future applications, it is necessary to have a larger sample size available that will allow us to perform inferential analyses optimally, calculate the effect size, and be able to allow generalization of the data obtained.

## Discussions

The gamification of modeling activities for learning purposes, as the experience done with PapyGame, has identified a set of phases that can be summarized as follows:

- definition of the learning paths;
- specification of the single learning exercises;
- definition of the checks/evaluation procedures and possible feedback;
- specification of the motivational elements and the respective game rules, e.g., how to assign points, bonuses, awards, and progress with levels.

Although we tried to cover all of them with PapyGame, there exists a number of practical issues that *make gamified education for modeling harder to realize*, as detailed in the remainder of this section.

In general, the definition of the learning paths does not require any specific tool to be handled, and it could even be drawn on paper. However, keeping these paths implicit makes them hard-coded in the modeling games, which decreases the chances of having shared gamified modeling scenarios. As a matter of fact, available gamification approaches for modeling adopt implicit paths, and there is no common view about suitable paths, also taking into account the learning objectives of the games.

The specification of the single learning exercise depends on the kind of game. With PapyGame, teachers could set up scenarios in which models are created from scratch or need to be completed with specific details (notably by creating properties, relationships, etc.). As a consequence, teachers are required to provide at least the expected initial and final versions of the models included in the exercises. Again, also in this case the specification of each game/exercise could be provided, e.g., in natural language, but this could make the definition ambiguous and would require an inspection of the evaluation procedures to completely understand the exercises. Similarly to the narratives, there is no shared repository of potential exercises, distinguishing, for example, the learning objectives and the corresponding initial/final models. From our personal experiences, we would mention here typical approaches for the exercises specification of the rework of models taken from literature and/or students’ own inputs.

Strictly related to the previous phase is the definition of checks and evaluation procedures. In fact, in the simplest scenario, the evaluation procedure would take as inputs the target model as provided by the teacher in the exercise definition together with the model produced by each student; if they match, then the exercise is successfully done. Otherwise, the exercise is failed. Nonetheless, some important challenges could arise when aiming at more advanced approaches. Notably, the mentioned matching technique would not work in those cases where students are required to propose their own modeling scenarios, since they are not defined in advance in the exercise. Similarly, it would be difficult to manage all those exercises in which more than one possible solution is viable. As a side observation, modeling languages are not necessarily executable; this in general is a problem for both students and teachers since it becomes more difficult to highlight certain problems (notably the correct use of UML diagrams) and provide self-guidance (trial and error). These practical challenges entail that both the kind of exercises and the corresponding feedback leave little chance to automation. In the current state of practice, a considerable amount of manual effort would be required for the teachers to evaluate advanced exercises and provide adequate feedback.

Analogously to the evaluation approach, also the game rules could range from straightforward in the simplest scenarios (e.g., the expected and delivered models need to be exactly the same) to complicated in case of advanced exercises. Notably, teachers might consider an exercise as passed if students reached a certain amount of completion, or the model satisfies pre-defined metrics. However, defining the different levels of completion could be very complicated, especially when to be checked automatically.

The students’ feedback, received during the experiments reported in Sect. 6, gives an advantage to PapyGame for the learning of modeling from a User Experience point of view. This advantage is independent of the fact that the students are regular players or not and we can consider this as further proof of the interest in gamification of software modeling.

However, there are still open issues that must be solved. The remainder of this section describes a set of future challenges we consider critical to make gamified modeling a more effective educational approach.

- *Adaptive and personalized feedback* Feedback plays a crucial role in students learning. The lack of feedback is one of the determining factors of student’s dropout [58]. The report of students’ work is considered a key element for quality in teaching [59]. In traditional educational settings, teachers provide feedback to students regarding their strengths and weaknesses. Unfortunately, this feedback process is more complicated than expected. According to [59], students have different perspectives on feedback processes, making it difficult to provide accurate feedback on performance in traditional classrooms. According to [58], in e-learning and gamification, it is possible to design tailored feedback that fits individual preferences. Therefore, future research activities should target personalized and adaptive feedback to provide adequate reports on students’ work, enhancing students’ performance and engagement levels.
- *Uncertain evaluation of modeling game tasks* In other gamification environments, validating that the user has accomplished a task is rather straightforward (e.g., imagine exercises to learn basic math operations). This is not the case for PapyGame where the task will involve the creation of modeling artefact. A pure syntactic comparison between that model and a sample solution is too restrictive as it would qualify as mistakes many models that would be semantically equivalent to the sample solution. A more precise evaluation could rely on a number of complementary techniques:
  - The characterization of the desired solution as a set of OCL constraints that any valid model should satisfy. There is a clear trade-off between the number of precision of the constraints and the degree of freedom of the models that satisfy them.
  - The use of clustering algorithms that aim to efficiently classify models in buckets based on a certain notion of structural similarity [60].
  - The combination of all of the above produces an uncertain correctness measure that should then be manually confirmed by the instructor (but that would at least automatically filter out those under a certain threshold).
- *Repository of modeling games* the design quality of the games is a critical element of the success of any gamification strategy. As such, we plan to start a public repository where educators and other professionals can upload and share the games they have used and the lessons learned from applying them in a teaching context. A specialized infrastructure for managing game models, e.g., inspired by [61], may be proposed, especially if game designs come with accompanying artifacts like sample models.
- *Personalized and cooperative learning* Gamification principles have proven to be very effective in motivating target users in keeping their engagement within everyday challenges, including dedication to education, use of public transportation, adoption of healthy habits, and so forth. School closures due to the COVID-19 pandemic and thus the sudden change in the management of the students’ educational pathways have uncovered the need for methods and digital systems able to support teachers in defining educational content and objectives for their classrooms and to keep students engaged in their training paths.
  Future investigations should be devoted to approaches, techniques, and tools to design and release cooperative learning paths. According to [62], the perception to be in a community plays a key role in the educational setting. The use of cooperative learning can be useful to bring out this sense of community, making gamification more effective despite individual differences, and including marginalized students. Our intention is to insert exercises carried out in groups and later competitions between classes.
  These approaches are expected to leverage AI techniques for adaptive gamification to support teachers in the process of defining and monitoring dedicated learning paths for their students. By generating dedicated learning paths and personalized feedback, these solutions are expected to facilitate learning, to encourage motivation and engagement, to improve students’ participation and cooperation, and to stimulate students to expand their knowledge.
- *Intelligent game adaptation* PapyGame allows the definition of games as sequences of mini-games. Our intention is to extend it with a more flexible scenario where this predefined sequence can be altered at runtime based on the monitoring of the personalized student learning process.
  This is especially useful in a multi-player scenario where we will have available monitoring data to make smarter decisions and reconduct (or at least suggest changes) the gamification based on the players’ behavior and results.
- *Desktop vs web-based client* using a “ heavy client” is not ideal in the case of academic learning. The installation of the software, of our PapyGame plugin, of the hardware and software compatibility (JVM) complicates the implementation of the gamification experience within the university courses (change of rooms, change of the place of the users of machines). We think that a web-based version of PapyGame could be more effective and solve many of the problems we encountered during the setup of our experiments with students.

## Conclusion

Software models are high value-added elements in a software project. The learning curve concerning modeling is unfortunately quite high and partly prevents a wide adoption of modeling in IT projects. An underlying reason for this problem lies in the fact that this learning process is threefold, since it concerns the very principle of modeling (abstraction, modularity, etc.), the notation, and the model editor. In this paper, we presented our work on gamifying this process to simplify it. Our primary objective was to experience and experiment this gamification with a real modeling environment and with real learners in order to evaluate the interest of such an approach. We chose Papyrus, the open-source reference environment for UML modeling in Eclipse. After explaining our choices regarding the target audience, the modeling dimensions to consider, and the gamification elements, we also defined an abstract architecture that could be used as a basis for implementation in other modeling environments. The feedback from the students from a UX perspective is very encouraging and shows the elements that made the difference. These results also show room for improvement (games, a more constrained environment, and a better management of technical issues) and point to ways to improve the overall approach to gamification software modeling.

## Footnotes

1 https://www.papygame.com.

2 https://www.eclipse.org/papyrus/.

3 https://ci.eclipse.org/papyrus/view/PapyGame/.

4 https://www.eclipse.org/papyrus/.

5 https://ci.eclipse.org/papyrus/view/PapyGame/.

6 https://github.com/google/gson.

7 https://github.com/ReactiveX/RxJava.

8 https://www.rstudio.com/.

9 https://osf.io/xjmp4.

10 https://www.jamovi.org/.
